# Effects of storage on mixed-culture biological electrodes

**DOI:** 10.1038/srep18433

**Published:** 2015-12-18

**Authors:** Soroush Saheb Alam, Frank Persson, Britt-Marie Wilén, Malte Hermansson, Oskar Modin

**Affiliations:** 1 Division of Water Environment Technology, Department of Civil and Environmental Engineering, Chalmers University of Technology, Gothenburg, Sweden; 2 Chemistry and Molecular Biology, University of Gothenburg, Gothenburg, Sweden

## Abstract

Storage methods are important to preserve the viability and biochemical characteristics of microbial cultures between experiments or during periods when bioreactors are inactive. Most of the research on storage has focused on isolates; however, there is an increasing interest in methods for mixed cultures, which are of relevance in environmental biotechnology. The purpose of this study was to investigate the effect of different storage methods on electrochemically active enrichment cultures. Acetate-oxidizing bioanodes generating a current density of about 5 A m^−2^ were enriched in a microbial electrolysis cell. The effect of five weeks of storage was evaluated using electrochemical techniques and microbial community analysis. Storage by refrigeration resulted in quicker re-activation than freezing in 10% glycerol, while the bioelectrochemical activity was entirely lost after storage using dehydration. The results showed that the bioelectrochemical activity of bioanodes stored at low temperature could be retained. However, during the re-activation period the bioanodes only recovered 75% of the current density generated before storage and the bacterial communities were different in composition and more diverse after storage than before.

Storage of microorganisms is essential in microbiology and biotechnology^1^. For example, researchers must be able to store microbial cultures between experiments and in industrial or environmental applications of biotechnology it is important to be able to maintain the viability and biochemical characteristics of specific strains or enrichment cultures during inactive periods.

Previous research on storage methods has mainly focused on pure cultures. Cryopreservation and drying methods are the most common and various techniques have been investigated^1^,^2^,^3^. It is generally accepted that a large fraction of the microbial cells will die during prolonged storage, but by having a high initial concentration (>10^7^ cell mL^−1^) some cells will survive and can be used to maintain the culture^2^. This is acceptable for pure cultures, but in the case of microbial consortia varying viability of different microorganisms during storage can affect the relative abundance of the different microorganisms and thereby the biochemical characteristics of the culture. In the field of environmental biotechnology, enrichment cultures are often used and several workers have pointed out that further research on preservation techniques for complex microbial consortia is needed^1^,^4^.

Recently, several studies have investigated storage of microbial mixed cultures of relevance in environmental biotechnology. For example, investigations of the effect of storage temperature on anaerobic sludge showed that anaerobic granules could be stored for 10 months at room temperature^5^ and storage of anaerobic sludge at room temperature or refrigerated conditions (4 °C) allowed faster re-activation than freezing (−20 °C) or freeze-drying^6^. For aerobic granular sludge, temperature was shown to have an important effect with storage at low temperature (4 °C or freezing) allowing better preservation of granule structure and activity than storage at room temperature^7^,^8^. Lv, *et al.*^9^ developed an acetone dehydration method for storage of aerobic granules. The granules were dried in acetone gradients to less than 1% water content. Upon re-activation, the granules could retain their organic degradation capacity in 12 hours. Wan, *et al.*^10^ showed that storage of aerobic granules for over one year was possible in deionized water, acetone, acetone/isoamyl acetate, and saline water, but not in formaldehyde solution. Heylen, *et al.*^4^ developed a cryopreservation method for anammox enrichment cultures using dimethyl sulfoxide as the cryoprotectant.

To our knowledge, there are no investigations of storage methods for electrochemically active enrichment cultures. Research on microbial electrochemical technologies has developed rapidly during the last 15 years^11^. The most well-known examples are the microbial fuel cell (MFC) and the microbial electrolysis cell (MEC), which both could be used to recover energy from wastewater organics. Microorganisms oxidize organic substances and transfer electrons to an anode. The electrons are circuited to the cathode where a reduction reaction takes place. In the MFC, oxygen is reduced and electrical energy can be harvested from the external circuit because of the favourable difference in redox potential between organics oxidation and oxygen reduction^12^. In the MEC, an electrical energy input is needed to drive the electrons to the cathode where hydrogen ions are reduced to hydrogen gas^13^. Other applications of microbial electrochemical technologies include e.g. recovery of various metals^14^, stimulation of the degradation of polycyclic aromatic hydrocarbons^15^, production of hydrogen peroxide^16^,^17^ and caustic^18^, recovery of ammonium^19^, and use as BOD- 20 or toxicity sensors^21^. Systems with biological cathodes have been used e.g. for denitrification^22^ or for reducing carbon dioxide to methane^23^ or acetate^24^,^25^.

Investigating storage of biological electrodes is relevant for several reasons. Researchers sometimes want to store electrochemically active enrichment cultures in between experimental runs and in engineering applications it may be relevant to store well-functioning biological electrodes during periods of inactivity. Biological electrodes are also significantly different from the other types of mixed microbial cultures that have previously been investigated in storage studies, i.e. anaerobic and aerobic granules, anaerobic sludge, and anammox cultures. Both the viability of the microorganisms involved and the structure of the biofilm formed on the electrode surface are very important for the bioelectrochemical activity. The bacteria must be able to establish electrical contact with the electrode, e.g. through nanowires or soluble shuttles^26^,^27^,^28^. If electrical contact is broken because the storage method e.g. results in a dead layer of biofilm near the electrode surface or causes structural damage to the biofilm, bioelectrochemical activity may be lost despite the presence of some viable bacteria. Thus, storage methods that have worked for e.g. granular sludge may not work for biological electrodes.

The goal of this study was, therefore, to investigate if storage of mixed-culture, acetate-oxidizing anodes was possible using three different methods that have previously been used to store other types of mixed microbial cultures. The investigated storage methods are (1) refrigeration at 4 °C, (2) freezing at −70 °C in 10% glycerol solution and (3) acetone dehydration. Refrigeration is a method that is easy to carry out and is assumed to preserve the culture by reducing the rate of the metabolic processes at low temperature. Freezing in 10% glycerol is a common method to store microbial cultures^1^. The low temperature halts all metabolic activities and the glycerol protects the cells from damage caused by the formation of ice crystals. The acetone dehydration method was developed by Lv, *et al.*^9^ and was shown to be a convenient method for storage of aerobic granules. For assessment of the effects of storage on the biological electrodes in this study, the activity before and after storage was analysed using current measurements and electrochemical tests. Furthermore, the bacterial community composition of the enrichment cultures was investigated before storage and after re-activation using high throughput amplicon sequencing of the V4 region of the 16S rRNA gene.

## Results

### Overall current production in the reactor

**Figure 1:**
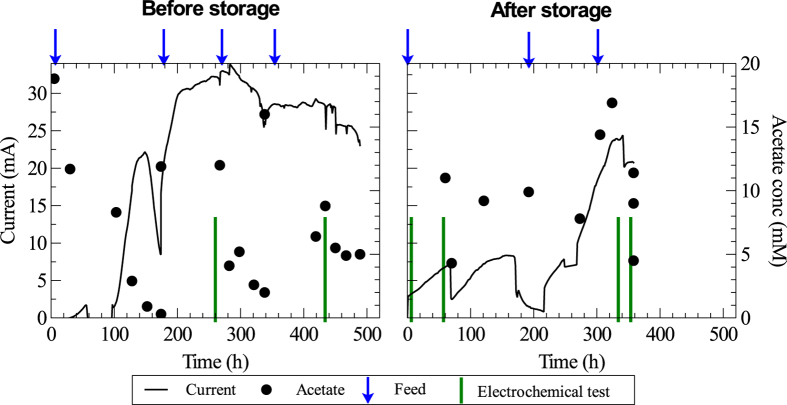
Current and acetate concentration with time in the reactor when the anodes were controlled at 0 V vs SHE. Arrows show when the nutrient medium was replaced and vertical lines show when normal operation was stopped and electrochemical tests were carried out.

Figure 1 shows the total current produced by the eight anodes in the MEC. Bioelectrochemical activity was observed after 100 hours with the current increasing up to about 22 mA in the following 48 hours. After that, there was a drop in current due to a decrease in the acetate concentration. The medium was replaced at this point. The current increased to 35 mA after 300 hours of operation and then reached a stable level of around 28 mA for the rest of the operation before storing the electrodes.

After five weeks of storage, the MEC was restarted. The total current produced in the MEC increased to around 8 mA after 154 hours with two temporary drops in current (Fig. 1). The first drop occurred because of electrochemical tests that were performed after 62 hours of operation. Electrochemical tests can have effect on the bioelectrochemical activity of the electrodes due to variation in potential applied to the cell during the tests^29^. The second drop after 154 hours occurred when the reference electrode was not working properly for a few hours. At around 330 hours after restarting the reactor, the current reached about 85% of the stable values generated before storage.

The acetate concentration was measured in samples taken from the reactor. During the first feeding cycle before storage (0–174 h), the current dropped significantly after the acetate concentration had decreased to 0.3 mM. During the rest of the experimental run, the nutrient medium was replaced before the acetate had decreased to concentrations that would cause severe drops in currents. In general, the acetate concentration was kept above 4 mM. Somewhat elevated acetate concentrations were measured in the reactor when samples were collected in association with electrochemical tests. The reason for this is unclear; however, acetate production by homoacetogens from hydrogen generated at the cathodes during the tests and insufficient mixing of the liquid in the reactor when the sample was taken are factors that could have contributed.

### Electrochemical tests

The effects of storage methods was investigated on duplicate electrodes, i.e. two anodes and two cathodes were stored using refrigeration, two using freezing, and two using acetone dehydration. At the time of storage, two anodes and two cathodes were also harvested for microbial community analysis and microscopy. These were replaced by new graphite rods when the reactor was restarted after storage. The bioelectrochemical activity of the electrodes was evaluated using cyclic voltammetry (CV) and linear sweep voltammetry (LSV). The anodes clearly showed bioelectrochemical activity by attached biofilms. The cathodes, on the other hand, did not appear to catalyze any bioelectrochemical reaction during operation of the reactor (see Supplementary Fig. S2 online); however, biofilm also developed on these electrodes. The cathode biofilms likely utilized the H_2_ gas that was abiotically generated on the cathode surfaces.

**Figure 2:**
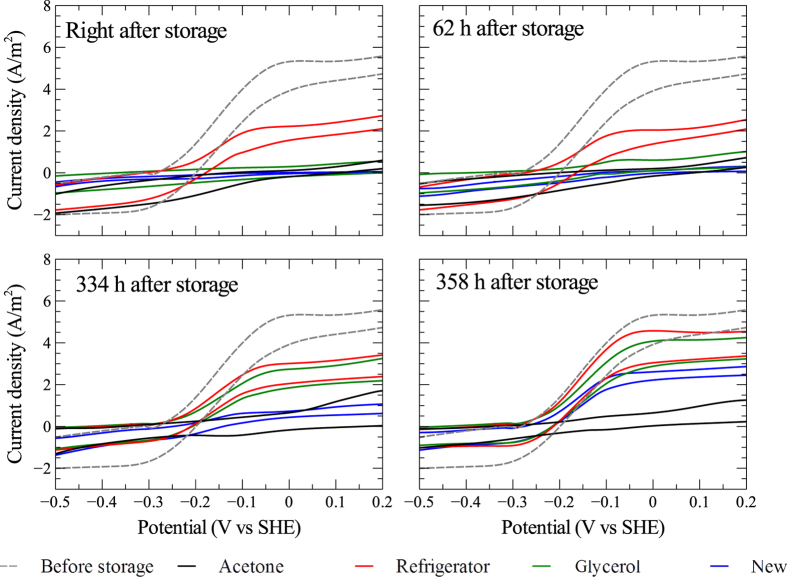
Cyclic voltammograms of anodes in the reactor. The dashed lines show the average cyclic voltammograms for all eight anodes in the reactor obtained during the second test before storage (see Fig. 1 for the times of these tests). The solid lines shows the average cyclic voltammograms for duplicate electrodes exposed to the three storage methods: acetone dehydration, refrigeration, and freezing in 10% glycerol solution. New refers to new graphite rod anodes placed in the reactor when the system was restarted after storage. The cyclic voltammetry was carried out between −1.0 and 0.5 V vs SHE; however, only the region between −0.5 V and 0.2 V is shown in the figure.

The results from the CV tests of the anodes are shown in Fig. 2. Before storage the anodes had a steep rise in current at about −0.17 V indicating that acetate was biologically oxidized. After storage, the anodes stored by refrigeration immediately showed biological activity, as seen by the rise in current at −0.17 V vs SHE. For the other stored anodes as well as the newly inserted anodes, no activity was observed until after 334 hours when also the anodes stored by glycerol-freezing showed bioelectrochemical response. The last test was done after 358 hours of operation since the restart. The results showed a similar pattern in CV curves for the anodes stored by refrigeration, glycerol-freezing, and the new anodes, with noticeable rises in current at −0.17 V vs SHE. However, the anodes stored using the acetone dehydration method did not show any bioelectrochemical activity during the whole re-activation period (see Supplementary Fig. S3–S5 online for the first derivative of the CV curves and the cyclic voltammograms for each individual anode in the reactor).

**Figure 3:**
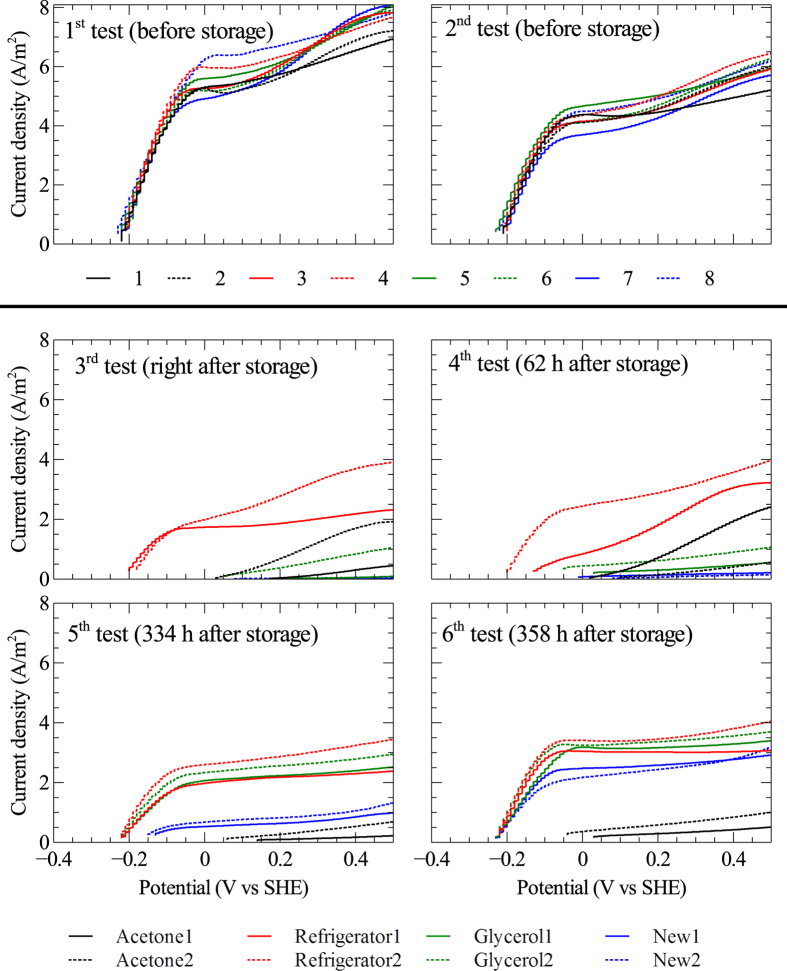
Linear sweep voltammograms from six different tests of the anodes in the reactor. Two tests (1^st^–2^nd^) were done before storage (see Fig. 1 for the times of these test) and the rest (3^rd^–6^th^) after storage. The figure legends refer to the three storage methods used: acetone dehydration, refrigeration, and freezing in 10% glycerol solution. New refers to new graphite rod anodes placed in the reactor when the system was restarted after storage.

Figure 3 shows the results for LSV tests of the anodes in the reactor. The tests before storage showed that all anodes had similar biological activity. Right after storage, different anodes exposed to different storage methods showed varied response to LSV. The anodes stored by refrigeration instantaneously showed biological activity, corresponding to 43 ± 3% (average value of duplicate electrodes) of the current measured at 0 V vs SHE before storage. At 62 hours of operation after storage, the anodes stored by the glycerol-freezing method showed some activity, corresponding to 7 ± 3% of the level before storage. After 334 hours of operation, the anodes stored by glycerol-freezing and refrigeration had increased their biological activity and reached 51 ± 7% of the level before storage. Current production by the new electrodes was also observed at this time. The final test, after 358 hours of operation, showed that the biological response of anodes stored by refrigeration and glycerol-freezing reached about 75 ±+ 4% of the level before storage, while the new anodes produced 54 ± 3% of the pre-storage current density. However, there was no significant progress observed for the electrodes stored using acetone dehydration. These electrodes produced, however, currents of about 2 A m^−2^ at a potential of about 0.2–0.4 V vs SHE in the 3^rd^ and 4^th^ LSV test, but these temporary increases in current occurred at a higher potential compared to the anodes before storage and are not likely caused by biological oxidation of acetate by microorganisms. Instead, abiotic oxidation of organic substances released by inactive biofilms on the surfaces of the electrodes may have been responsible for the observed increases in current at these particular occasions.

**Figure 4:**
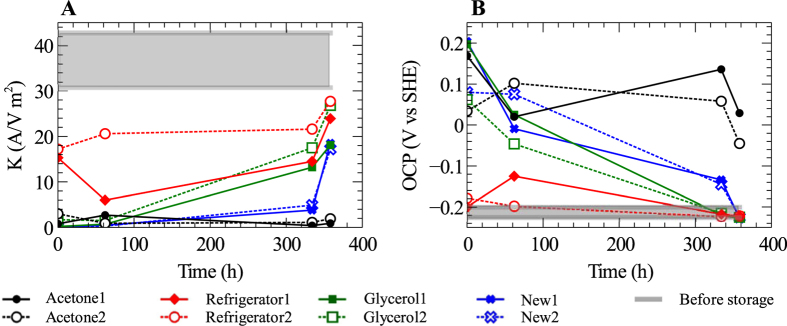
(**A**) Kinetic factor (K), which corresponds to i_0_F/RT, for different tests after storage. (**B**) Open circuit potential (OCP) of the eight different anodes placed in the reactor after storage. The grey regions show the variation in K and OCP of the eight anodes before storage. The figure legends refer to the three storage methods used: acetone dehydration, refrigeration, and freezing in 10% glycerol solution. New refers to new graphite rod anodes placed in the reactor when the system was restarted after storage.

The bioelectrochemical activity of the anodes was also evaluated using the open circuit potential (OCP) and the kinetic factor (K), which is a measure of the kinetic facility of electrode reactions (see Supplementary Methods online) (Fig. 4). After storage, K dropped for all storage methods; however, the anodes stored using refrigeration all had a significantly larger K than the anodes stored in other ways. K of the new anodes were around 135 times lower than those stored by refrigeration right after storage (Fig. 4A). This shows the higher catalytic ability of the anodes stored by refrigeration in comparison to bare graphite rods. In the test after 358 hours of re-activation, the anodes stored using refrigeration, glycerol-freezing, and the new anodes had reached kinetic factors of about 16–28 A V^−1^ m^−2^, whereas those exposed to the acetone-dehydration method still showed a low K of about 1 A V^−1^ m^−2^.

The acetate/CO_2_ redox couple has a standard reduction potential of −0.28 V and a biological anode oxidizing acetate would have an OCP near this value. Before storage the OCP was around −0.21 V vs SHE for all anodes (Fig. 4B). The anodes stored by refrigration showed biological activity directly after storage. The OCP of the anodes stored by glycerol-freezing reached −0.22 V vs SHE 334 hours of operation after storage. The new anodes reached −0.22 V vs SHE 358 hours of operation after storage, while the anodes dehydrated in acetone never recovered their original low OCP.

### Microbial community analysis

Scanning electron microscopy (SEM) showed that all anodes were covered by a thick layer of biofilm whereas the cathodes were covered by a thinner layer of microbial cells (see Supplementary Fig. S6 online). The biofilms on the electrodes were investigated with Illumina MiSeq 16S rRNA amplicon sequencing of the whole bacterial community. Biofilm samples were collected after 489 hours of operation of the reactor prior to storage and after the 358-hour re-activation period. The diversity of the anode- and cathode microbial communities was higher for the samples after storage and for the new electrodes, compared to the anodes and cathodes before storage, as indicated by the OTU richness and the inverse Simpson diversity index (see Supplementary Table S1 online).

To visualize differences between the microbial communities, ordination by non-metric multidimensional scaling (NMDS) on distances based on You-Clayton dissimilarities was used (see Supplementary Fig. S7 online). The microbial communities on the anodes before storage and after the different storage methods separated in the ordination. Likewise, the microbial communities on the cathode before storage and the new cathode after storage separated from each other and from the anode communities, indicating quantitative differences between the investigated microbial communities.

**Figure 5:**
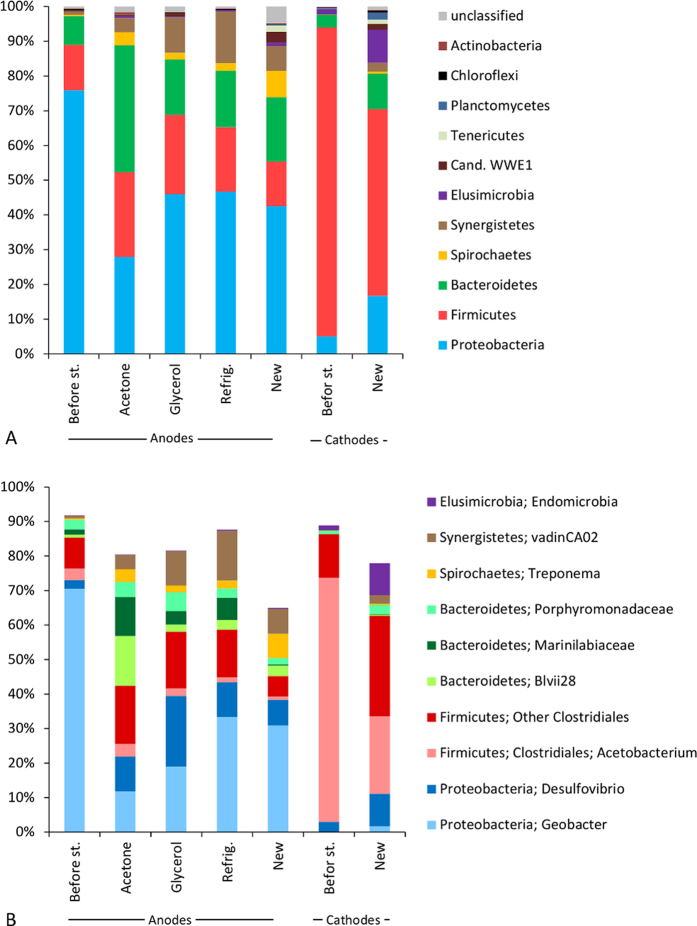
Relative abundance of 16S rRNA gene sequences in the electrode biofilms. (**A**) Distribution of phyla with relative abundance >0.5% in any of the samples. (**B**) Distribution of major taxa (relative abundance >5% in any of the samples) at highest possible taxonomic determination using the Greengenes taxonomy.

The most abundant sequences were affiliated to the phyla *Proteobacteria, Firmicutes, Bacteroidetes* and *Synergistetes*, with some additional phyla present in lower relative abundance (Fig. 5A). In total, 28 bacterial phyla were observed in the anode biofilms and 27 in the cathode biofilms. Many of the phyla contained one or a few dominant OTUs assigned to a particular genus or family (Fig. 5B). Among *Proteobacteria*, the major contributors were OTUs within *Geobacter* sp. and *Desulfovibrio* sp. Within *Firmicutes*, OTUs belonging to *Clostridiales* were dominating, with many OTUs affiliated to *Acetobacterium* sp. Also the phyla *Spirochaetes* and *Synergistetes* had major OTUs, *Treponema* sp. and the vadinCA02 genus within *Synergistaceae*, respectively.

## Discussion

The electrochemical tests showed that all the eight anodes in the MEC performed almost identically before storage (e.g. Fig. 3), which suggests they can be considered as replicates regardless of being placed in different positions within the reactor. This means that the microbial community on the anode harvested for analysis before storage could likely be considered as representing the community on all anodes in the reactor. Before storage, the current density was around 5 A m^−2^ at 0 V vs SHE. This is comparable to a previous study by Torres, *et al.*^30^, which reported current densities of about 3 A m^−2^ generated by graphite rod anodes enriched at a potential of +0.02 V vs SHE with acetate-containing nutrient medium. They also showed that higher current density, reaching 10.3 A m^−2^, could be obtained with anodes enriched at −0.15 V vs SHE.

The bacterial community on the anodes prior to storage was dominated by *Geobacter* sp. which made up 71% of all sequences in the anode biofilms. *Geobacter* species are well-known for their ability of extracellular electron transfer via pili (nanowires)^31^. In microbial electrochemistry, *G. sulfurreducens* has received particular attention, due to their efficient direct electron transfer to anodes^32^ resulting in high power output and high columbic efficiency in MFCs^33^. Predominance of *Geobacter* sp. has previously been observed in anode biofilms of acetate-fed MFCs^34^,^35^ and in MECs with mixed culture inoculum operated at anode potentials in the range of −0.15 to 0.02 V vs SHE^30^. The enrichment of *Geobacter* sp. on the anodes in this study further confirms that this type of bacteria are highly selected for at anode potentials of around 0 V vs SHE and lower.

A microbial community, clearly different from the anode communities, developed on the cathode surfaces (see Fig. 5 and Supplementary Fig. S7 online). There was a high abundance of *Acetobacterium* sp., which are capable of oxidizing H_2_ and producing acetate^36^. *Clostridiales* and *Desulfovibrio* sp. were also present at the cathode and anode biofilms at considerable relative abundances. Interestingly, several acetogenic *Clostridium* species^24^ as well as *Desulfovibrio* species^37^ have been shown to directly accept electrons from cathodes producing organics acids or H_2_. Although CV tests indicated that the cathode biofilms contained redox-active components, there was no evidence that the biofilms were able to grow on electrons directly harvested from the cathodes (see Supplementary Fig. S2 online). Instead, it seems likely that the bacteria present on the cathodes mainly utilized the abiotically generated H_2_. This has been shown for *Desulfovibrio* species, which can use hydrogen as electron donor, e.g. when being supplied with acetate as carbon source^38^.

After five weeks of storage of the bioelectrochemically active anodes, results from the different electrochemical tests showed that the anodes stored using refrigeration could revive faster than the anodes stored using other methods. Indeed, immediately after storage, the refrigerator-stored anodes showed bioelectrochemical response in the CV and LSV tests (Figs 2 and 3), had open-circuit potentials similar to before storage and kinetic factors about half of pre-storage levels (Fig. 4). Anodes stored by refrigeration and glycerol-freezing both showed higher electrochemical activity than new anodes that were installed in the reactor. This shows that the surfaces of the anodes stored by refrigeration and glycerol-freezing both had some viable biofilms which could be revived after storage. This biofilm probably served as inoculum for the new electrodes placed in the reactor after storage. The acetone dehydration method has been shown to be suitable for storage of aerobic granular sludge^9^; however, it appears to be unsuitable for storage of biological anodes since the bioelectrochemical activity was completely destroyed and could not be revived during the 358 hours re-activation period after storage. The SEM micrographs showed that also the anodes stored using acetone dehydration had a thick biofilm layer at the end of the study. The reason that this biofilm was not electrochemically active could be a dead layer of biofilm on the surface of the electrodes, which would prevent a new biofilm from establishing electrical contact with the electrode surface.

The microbial analysis results showed that the microbial communities that developed on the stored anodes after 358 hours of re-activation were distinctly different from the microbial community on the anodes prior to storage (see Supplementary Fig. S7 online). In particular, the relative abundance of *Geobacter* sp. decreased and more diverse communities developed (see Fig. 5 and Supplementary Table S1 online) with higher relative abundance of sequences belonging to the phyla *Bacteroidetes, Synergistetes*, and *Spirochaetes* as well as *Desulfovibrio* sp. within *Proteobacteria* and *Clostridiales* within *Firmicutes* (Fig. 5). Although there are several representatives of electrogenic bacteria among these taxonomic groups^39^, the bioelectrochemical activity of the stored anodes was lower than on the anodes prior to storage. In mixed inoculum anode microbial communities, the relative *Geobacter* abundance was previously shown to reflect bioelectrochemical activity^30^,^40^, which indicate the particular efficiency of this bacterium for electron transfer.

A reason for the increased microbial diversity in the reactor after storage could be a higher availability of alternative organic substrates caused by an increased amount of dead biomass on the electrodes. All the storage methods likely killed some of the microbial cells on the anodes. Before storage, bacteria capable of oxidizing acetate with the anode as electron donor (e.g. *Geobacter* sp.) were highly selected for in the reactor. After storage, many of these bacteria were probably killed and other bacteria capable of using material released from the dead biomass would have opportunity to grow and increase in relative abundance. *Desulfovibrio* sp. increased in relative abundance on all electrodes after storage. Since they increased also on the cathode and the acetone-treated anode, which lacked bioelectrochemical activity, they were probably not highly important for current generation on the anode. Instead, it appears that these bacteria could survive the storage methods and grow in the reactor using fermentation or sulfate as electron acceptor^38^.

One open question is why the MEC did not regain its previous performance after the storage period. Not even the new anodes installed after the storage period reached the bioelectrical activity of the anodes prior to storage. The microbial community both on the new anodes and the new cathode was also clearly different with a higher diversity and a different composition. The differences may partly be explained by the shorter operation time available for selection of the new electrode specific communities (358 h vs 489 h). Surprisingly, this did not lead to a more uniform community. Different inocula have resulted in different performance of MFCs^41^,^42^, indicating that the founding bacterial community is important for the process performance. However, convergence of microbial communities and similar production of voltage and power density of MFCs with different inocula have indicated that good performance and selection of the desired microorganisms can be independent of the inoculum source at long term operation (two months)^35^. It should also be noted that considerable temporal dynamics of electrode microbial communities has been described even after long term operation, for anodes^43^ and cathodes^44^, presumably shifting with changes in functional activity. Hence, it is not too surprising that the microbial communities developing on the new electrodes were different in composition compared to the ones prior to storage.

In conclusion, refrigeration allowed the best retention of bioelectrochemical activity of the tested methods. However, pre-storage levels could not be recovered during the re-activation period. This has different implications depending on the purpose of storage:

- For a start-up of a new reactor, it is faster to enrich a new high-performing biological anode from a wastewater inoculum than to re-use a biological anode stored using any of the methods tested in this study.
- For demonstration purposes, storage using refrigeration is satisfactory since bioelectrochemical activity was observed immediately upon re-start of the reactor.
- For a researcher who wishes to preserve a specific microbial community composition for future experiments, one has to realize that storage methods exert a selection pressure on the biological electrodes. The methods tested here all resulted in a more diverse microbial communities after storage. Further studies should also investigate storage under strictly anaerobic conditions. In this study the electrodes were exposed to air, which may have killed some oxygen-sensitive species and acted as a selection pressure.

It is also clear from this study that storage by acetone dehydration, which is a type of drying method, is not suitable for biological anodes. Drying methods are very common for long-term storage of microbial cultures; however, they can result in large loss of viability depending on the type of microorganism and its growth state^2^. It seems likely that drying affects the structure of the biofilm, which could destroy electrical connections to the anode. In this study, acetone dehydration caused complete loss of bioelectrochemical activity, which could not be recovered during the 358 h re-activation period. This suggests that drying resulted in an inactive biomass layer on the anode surface, which efficiently prevented the establishment of a new electrochemically-active biofilm. In this study, acetone-dehydration was investigated because it is a simple and convenient method that does not require expensive equipment, and it was shown to be successful for aerobic granules^9^. However, the effect of other dehydration methods, such as freeze-drying, should be investigated in further studies.

## Materials and Methods

### Description of the reactor and its operation

A plexiglas single-chamber microbial electrolysis cell (MEC) (10 × 10 × 6 cm^3^) containing 16 graphite rod electrodes, which each had a surface area of 11.3 cm^2^, was investigated. Eight of the electrodes were used as anodes and eight were used as cathodes. The reactor also contained an Ag/AgCl reference electrode with an offset of 0.197 V vs SHE. The total volume of the plexiglas chamber was 650 mL. A nutrient medium with a total volume of 1 L was circulated through the reactor from an external 0.5 L bottle at a flow rate of 1.35 mL s^−1^. A schematic of the experimental setup is shown in Supplementary Fig. S1 online. At the start of the experiment, the reactor was inoculated with 100 mL of raw municipal wastewater. The nutrient medium contained acetate, mineral salts, and trace elements (see Supplementary Methods online). The reactor was operated in fed-batch mode and the nutrient medium was replaced regularly as acetate was consumed.

The reactor was operated by controlling the anode potential at 0 V vs SHE. After 489 hours of operation, the electrodes were stored for five weeks using three different storage methods: (1) storage by submerging the electrodes in nutrient medium in a refrigerator (+4 °C), (2) storage by submerging the electrodes in 10% glycerol solution followed by freezing at −70 °C, and (3) acetone dehydration followed by storage at room temperature according to Lv, *et al.*^9^. Each storage method was evaluated using duplicate anodes and two anodes were also harvested for microscopy and microbial community analysis. During storage and when the electrodes were handled outside the reactor, they were exposed to air.

Prior to restarting the reactor, the anodes stored using refrigeration and freezing were kept at room temperature for about 2 hours. Glycerol was washed away from the freezer-stored anodes by submerging them in milli-Q water for about 30 s. Then, all anodes were again placed in the reactor and incubated at 0 V vs SHE for 358 hours. The two anodes that had been harvested for microscopy and microbial community analysis at the time of storage were replaced by two new graphite rod anodes inserted into the reactor. At the end of the experiment, all electrodes were collected for microscopy and microbial community analysis. The harvested electrodes were kept at −70 °C prior to analysis.

### Analytical methods

The acetate concentration was analysed using a high performance liquid chromatograph (HPLC) equipped with a UV detector. An Aminex HPX-87H column (BioRad) and a 5 mM H_2_SO_4_ eluent pumped at 0.5 mL min^−1^ were used. The pH was measured by a pH sensor and was around 7 during the whole experiment. During normal reactor operation, the anode potential was controlled using a potentiostat and the current was recorded using a data acquisition unit. The bioelectrochemical activity of the electrodes in the reactor was investigated on several occasions using cyclic voltammetry (CV) and linear sweep voltammetry (LSV). CV was swept between 0.5 and −1.0 V vs SHE at a scan rate of 5 mV s^−1^. LSV swept from open circuit potential to 0.5 V vs SHE at a scan rate of 1 mV s^−1^. Scanning electron microscopy was carried out as described in the Supplementary Methods online.

### Microbial community analysis

The biofilm on the electrodes was scraped off the surfaces using a sterile scapula and was homogenized in 15 ml sterile water using a Bagmixer 100 Minimix (Interscience). For DNA extraction, subsamples of the biofilm suspensions were used. DNA was extracted using the FastDNA spin kit for soil (MP biomedicals). The DNA concentration was measured using a NanoDrop ND-1000 spectrophotometer (Thermo Scientific) and the DNA extracts were diluted to 20 ng μl^−1^ with sterile water. PCR, next-generation sequencing, and bioinformatics analysis was carried out as described in the Supplementary Methods online.

## Additional Information

**How to cite this article**: Alam, S. S. *et al.* Effects of storage on mixed-culture biological electrodes. *Sci. Rep.* **5**, 18433; doi: 10.1038/srep18433 (2015).

## Supplementary Material

Supplementary Information

## References

[b1] Prakash O., Nimonkar Y. & Shouche Y. S. Practice and prospects of microbial preservation. FEMS microbiology letters 339, 1–9, doi: 10.1111/1574-6968.12034 (2013). 23083094

[b2] Morgan C. A., Herman N., White P. A. & Vesey G. Preservation of micro-organisms by drying; a review. Journal of microbiological methods 66, 183–193, doi: 10.1016/j.mimet.2006.02.017 (2006). 16632005

[b3] Suslow T. V. & Schroth M. N. Bacterial culture preservation in frozen and dry-film methylcellulose. Applied and Environmental Microbiology 42, 872–877 (1981). 16345889 10.1128/aem.42.5.872-877.1981 PMC244121

[b4] Heylen K., Ettwig K., Hu Z., Jetten M. & Kartal B. Rapid and simple cryopreservation of anaerobic ammonium-oxidizing bacteria. Applied and Environmental Microbiology 78, 3010–3013, doi: 10.1128/AEM.07501-11 (2012). 22307300 PMC3318846

[b5] Bae B.-U., Shin H.-S., Paik B.-C. & Chung J.-C. Re-activation characteristics of preserved anaerobic granular sludge. Bioresource Technology 53, 231–235 (1995).

[b6] Castro H., Queirolo M., Quevedo M. & Muxi L. Preservation methods for the storage of anaerobic sludges. Biotechnology Letters 24, 329–333, doi: 10.1023/A:1014080526608 (2002).

[b7] Gao D., Yuan X. & Liang H. Reactivation performance of aerobic granules under different storage strategies. Water Research 46, 3315–3322, doi: 10.1016/j.watres.2012.03.045 (2012). 22542063

[b8] Adav S. S., Lee D. J. & Tay J. H. Activity and structure of stored aerobic granules. Environmental Technology 28, 1227–1235, doi: 10.1080/09593332808618883 (2007). 18290532

[b9] Lv Y. *et al.* Drying and re-cultivation of aerobic granules. Bioresource Technology 129, 700–703, doi: 10.1016/j.biortech.2012.12.178 (2013). 23357589

[b10] Wan C., Zhang Q., Lee D. J., Wang Y. & Li J. Long-term storage of aerobic granules in liquid media: viable but non-culturable status. Bioresource Technology 166, 464–470, doi: 10.1016/j.biortech.2014.05.091 (2014). 24950091

[b11] Modin O. & Gustavsson D. J. Opportunities for microbial electrochemistry in municipal wastewater treatment - an overview. Water Science and Technology 69, 1359–1372, doi: 10.2166/wst.2014.052 (2014). 24718325

[b12] Logan B. E. *et al.* Microbial fuel cells: Methodology and technology. Environmental Science & Technology 40, 5181–5192 (2006). 16999087 10.1021/es0605016

[b13] Liu H., Grot S. & Logan B. E. Electrochemically assisted microbial production of hydrogen from acetate. Environmental Science & Technology 39, 4317–4320 (2005). 15984815 10.1021/es050244p

[b14] Modin O., Wang X., Wu X., Rauch S. & Fedje K. K. Bioelectrochemical recovery of Cu, Pb, Cd, and Zn from dilute solutions. Journal of Hazardous Materials 235–236, 291–297, doi: 10.1016/j.jhazmat.2012.07.058 (2012). 22910451

[b15] Yan Z., Jiang H., Cai H., Zhou Y. & Krumholz L. R. Complex Interactions Between the Macrophyte Acorus Calamus and Microbial Fuel Cells During Pyrene and Benzo[a]Pyrene Degradation in Sediments. Scientific Reports 5, 10709, doi: 10.1038/srep10709 (2015). 26023748 PMC4448661

[b16] Rozendal R. A., Leone E., Keller J. & Rabaey K. Efficient hydrogen peroxide generation from organic matter in a bioelectrochemical system. Electrochemistry Communications 11, 1752–1755, doi: 10.1016/j.elecom.2009.07.008 (2009).

[b17] Modin O. & Fukushi K. Production of high concentrations of H2O2 in a bioelectrochemical reactor fed with real municipal wastewater. Environmental Technology 34, 2737–2742, doi: 10.1080/09593330.2013.788041 (2013). 24527636

[b18] Rabaey K., Butzer S., Brown S., Keller J. & Rozendal R. A. High current generation coupled to caustic production using a lamellar bioelectrochemical system. Environmental Science & Technology 44, 4315–4321 (2010). 20446659 10.1021/es9037963

[b19] Wu X. & Modin O. Ammonium recovery from reject water combined with hydrogen production in a bioelectrochemical reactor. Bioresource Technology 146, 530–536, doi: 10.1016/j.biortech.2013.07.130 (2013). 23973971

[b20] Modin O. & Wilén B.-M. A novel bioelectrochemical BOD sensor operating with voltage input. Water Research 46, 6113–6120 (2012). 23021520 10.1016/j.watres.2012.08.042

[b21] Stein N. E., Hamelers H. V. & Buisman C. N. Stabilizing the baseline current of a microbial fuel cell-based biosensor through overpotential control under non-toxic conditions. Bioelectrochemistry 78, 87–91, doi: 10.1016/j.bioelechem.2009.09.009 (2010). 19896420

[b22] Clauwaert P. *et al.* Biological denitrification in microbial fuel cells. Environmental Science & Technology 41, 3354–3360 (2007). 17539549 10.1021/es062580r

[b23] Cheng S., Xing D., Call D. F. & Logan B. E. Direct biological conversion of electrical current into methane by electromethanogenesis. Environmental Science & Technology 43, 3953–3958 (2009). 19544913 10.1021/es803531g

[b24] Nevin K. P. *et al.* Electrosynthesis of Organic Compounds from Carbon Dioxide Is Catalyzed by a Diversity of Acetogenic Microorganisms. Applied and Environmental Microbiology 77, 2882–2886, doi: 10.1128/aem.02642-10 (2011). 21378039 PMC3126412

[b25] Marshall C. W., Ross D. E., Fichot E. B., Norman R. S. & May H. D. Electrosynthesis of commodity chemicals by an autotrophic microbial community. Applied and Environmental Microbiology 78, 8412–8420, doi: 10.1128/AEM.02401-12 (2012). 23001672 PMC3497389

[b26] Reguera G. *et al.* Extracellular electron transfer via microbial nanowires. Nature 435, 1098–1101, doi: 10.1038/nature03661 (2005). 15973408

[b27] Borole A. P. *et al.* Electroactive biofilms: Current status and future research needs. Energy & Environmental Science 4, 4813–4834, doi: 10.1039/c1ee02511b (2011).

[b28] Richter H. *et al.* Cyclic voltammetry of biofilms of wild type and mutant Geobacter sulfurreducens on fuel cell anodes indicates possible roles of OmcB, OmcZ, type IV pili, and protons in extracellular electron transfer. Energy & Environmental Science 2, 506–516, doi: 10.1039/b816647a (2009).

[b29] Yan H. J., Saito T. & Regan J. M. Nitrogen removal in a single-chamber microbial fuel cell with nitrifying biofilm enriched at the air cathode. Water Research 46, 2215–2224, doi: 10.1016/j.watres.2012.01.050 (2012). 22386083

[b30] Torres C. I. *et al.* Selecting Anode-Respiring Bacteria Based on Anode Potential: Phylogenetic, Electrochemical, and Microscopic Characterization. Environmental Science & Technology 43, 9519–9524, doi: 10.1021/Es902165y (2009). 20000550

[b31] Lovley D. R. *et al.* Geobacter: The Microbe Electric's Physiology, Ecology, and Practical Applications. Adv Microb Physiol 59, 1–100, doi: 10.1016/B978-0-12-387661-4.00004-5 (2011). 22114840

[b32] Bond D. R. & Lovley D. R. Electricity production by Geobacter sulfurreducens attached to electrodes. Applied and Environmental Microbiology 69, 1548–1555, doi: 10.1128/aem.69.3.1548-1555.2003 (2003). 12620842 PMC150094

[b33] Nevin K. P. *et al.* Power output and columbic efficiencies from biofilms of Geobacter sulfurreducens comparable to mixed community microbial fuel cells. Environmental Microbiology 10, 2505–2514, doi: 10.1111/j.1462-2920.2008.01675.x (2008). 18564184

[b34] Chae K. J., Choi M. J., Lee J. W., Kim K. Y. & Kim I. S. Effect of different substrates on the performance, bacterial diversity, and bacterial viability in microbial fuel cells. Bioresour Technol 100, 3518–3525, doi: 10.1016/j.biortech.2009.02.065 (2009). 19345574

[b35] Yates M. D. *et al.* Convergent development of anodic bacterial communities in microbial fuel cells. Isme J 6, 2002–2013, doi: 10.1038/ismej.2012.42 (2012). 22572637 PMC3475369

[b36] Balch W. E., Schoberth S., Tanner R. S. & Wolfe R. S. Acetobacterium, a New Genus of Hydrogen-Oxidizing, Carbon Dioxide-Reducing, Anaerobic Bacteria. International Journal of Systematic Bacteriology 27, 355–361 (1977).

[b37] Aulenta F., Catapano L., Snip L., Villano M. & Majone M. Linking bacterial metabolism to graphite cathodes: electrochemical insights into the H(2) -producing capability of Desulfovibrio sp. ChemSusChem 5, 1080–1085, doi: 10.1002/cssc.201100720 (2012). 22581429

[b38] Basso O., Caumette P. & Magot M. Desulfovibrio putealis sp. nov., a novel sulfate-reducing bacterium isolated from a deep subsurface aquifer. International journal of systematic and evolutionary microbiology 55, 101–104, doi: 10.1099/ijs.0.63303-0 (2005). 15653861

[b39] Sydow A., Krieg T., Mayer F., Schrader J. & Holtmann D. Electroactive bacteria-molecular mechanisms and genetic tools. Applied microbiology and biotechnology, doi: 10.1007/s00253-014-6005-z (2014). 25139447

[b40] Kiely P. D. *et al.* Anode microbial communities produced by changing from microbial fuel cell to microbial electrolysis cell operation using two different wastewaters. Bioresour Technol 102, 388–394, doi: 10.1016/j.biortech.2010.05.019 (2011). 20554197

[b41] Ieropoulos I., Winfield J. & Greenman J. Effects of flow-rate, inoculum and time on the internal resistance of microbial fuel cells. Bioresour Technol 101, 3520–3525, doi: 10.1016/j.biortech.2009.12.108 (2010). 20100658

[b42] Jiang D., Li B., Jia W. & Lei Y. Effect of inoculum types on bacterial adhesion and power production in microbial fuel cells. Applied biochemistry and biotechnology 160, 182–196, doi: 10.1007/s12010-009-8541-z (2010). 19214793

[b43] Ishii S. *et al.* Identifying the microbial communities and operational conditions for optimized wastewater treatment in microbial fuel cells. Water Research 47, 7120–7130, doi: 10.1016/j.watres.2013.07.048 (2013). 24183402

[b44] Marshall C. W., Ross D. E., Fichot E. B., Norman R. S. & May H. D. Long-term operation of microbial electrosynthesis systems improves acetate production by autotrophic microbiomes. Environ Sci Technol 47, 6023–6029, doi: 10.1021/es400341b (2013). 23676111

