# Supplementary material for: Effects of storage on mixed-culture biological electrodes
Source: Sci Rep. 2015 Dec 18;5:18433. doi: 10.1038/srep18433 (PMC4683449; doi:10.1038/srep18433)
Supplement: Supplementary Information [file srep18433-s1.pdf]

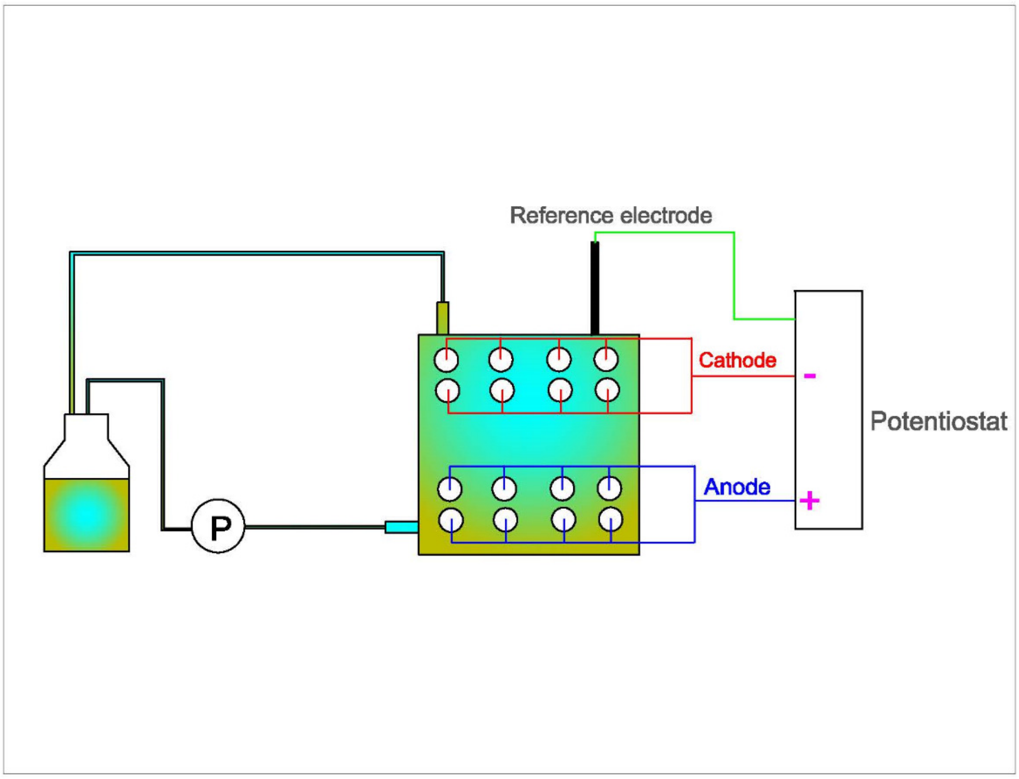

Figure S1. Schematic of experimental setup**.**

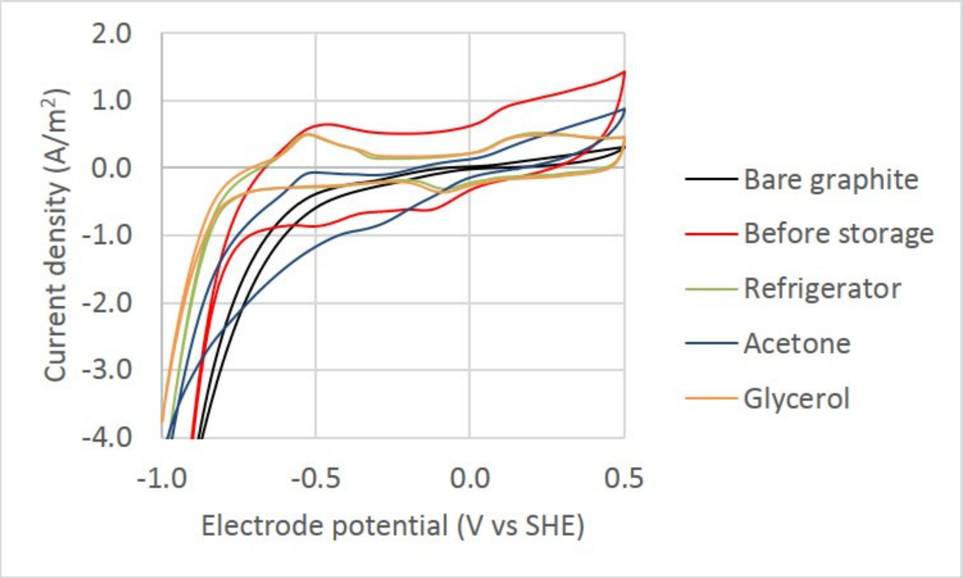

Figure S2. Cyclic voltammograms of some of the cathodes in the reactor. The lines show results for a bare graphite rod in the reactor and biofilm-covered cathodes taken before storage and right after storage using refrigeration, acetone dehydration, and glycerol-freezing. Comparison of the lines show that the biofilm-covered cathodes have greater hysteresis and show small oxidation and reduction peak indicating the presence of redox-active components in the biofilms while the voltammogram for the bare graphite rod is smooth and have low hysteresis. However, for the bare graphite the rapid rise in cathodic current occurs at higher electrode potential than for the biofilm-covered electrodes, which suggests that the biofilms did not catalyze hydrogen generation during normal operation of the reactor. Instead, the biofilm appears to have shifted the hydrogen generation potential slightly in the negative direction.

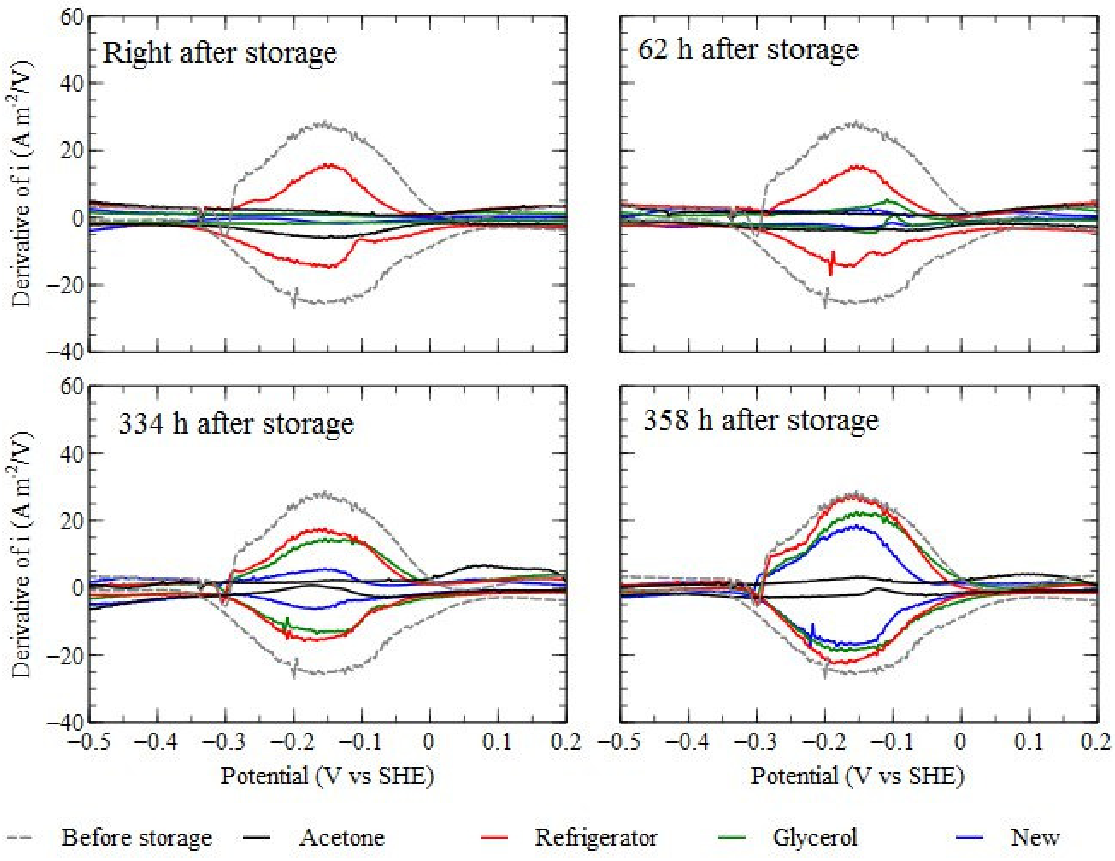

Figure S3. Derivative of cyclic voltammograms of the biological anodes. The dashed grey line shows the derivative of an average of cyclic voltammograms carried out for the eight anodes during the second test before storage. The solid lines shows the average of duplicate electrodes. The figure legends refer to the three storage methods used: acetone dehydration, refrigeration, and freezing in 10% glycerol solution. New refers to new graphite rod anodes placed in the reactor when the system was restarted after storage. The cyclic voltammetry was carried out between -1.0 and 0.5 V vs SHE; however, only the region between -0.5 V and 0.2 V is shown in the figure.

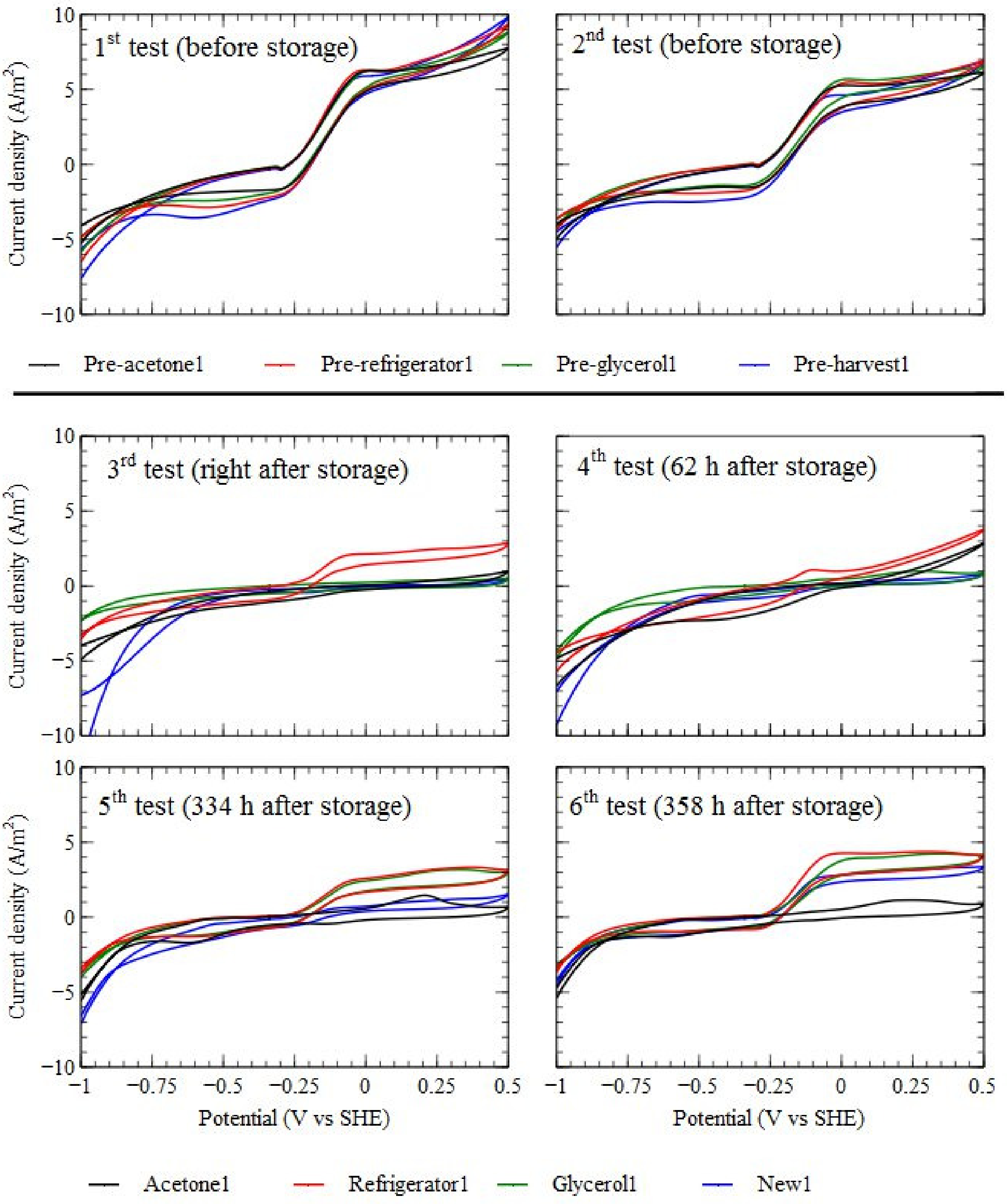

Figure S4. Cyclic voltammograms from six different tests of the anodes in the reactor. Two tests (1st-2nd) were done before storage and the rest (3rd-6th) after storage. The figure legends refer to the three storage methods used: acetone dehydration, refrigeration, and freezing in 10% glycerol solution. New refers to new graphite rod anodes placed in the reactor when the system was restarted after storage. This graph shows results from four of the eight anodes placed in the reactor (Figure S5 shows the other four).

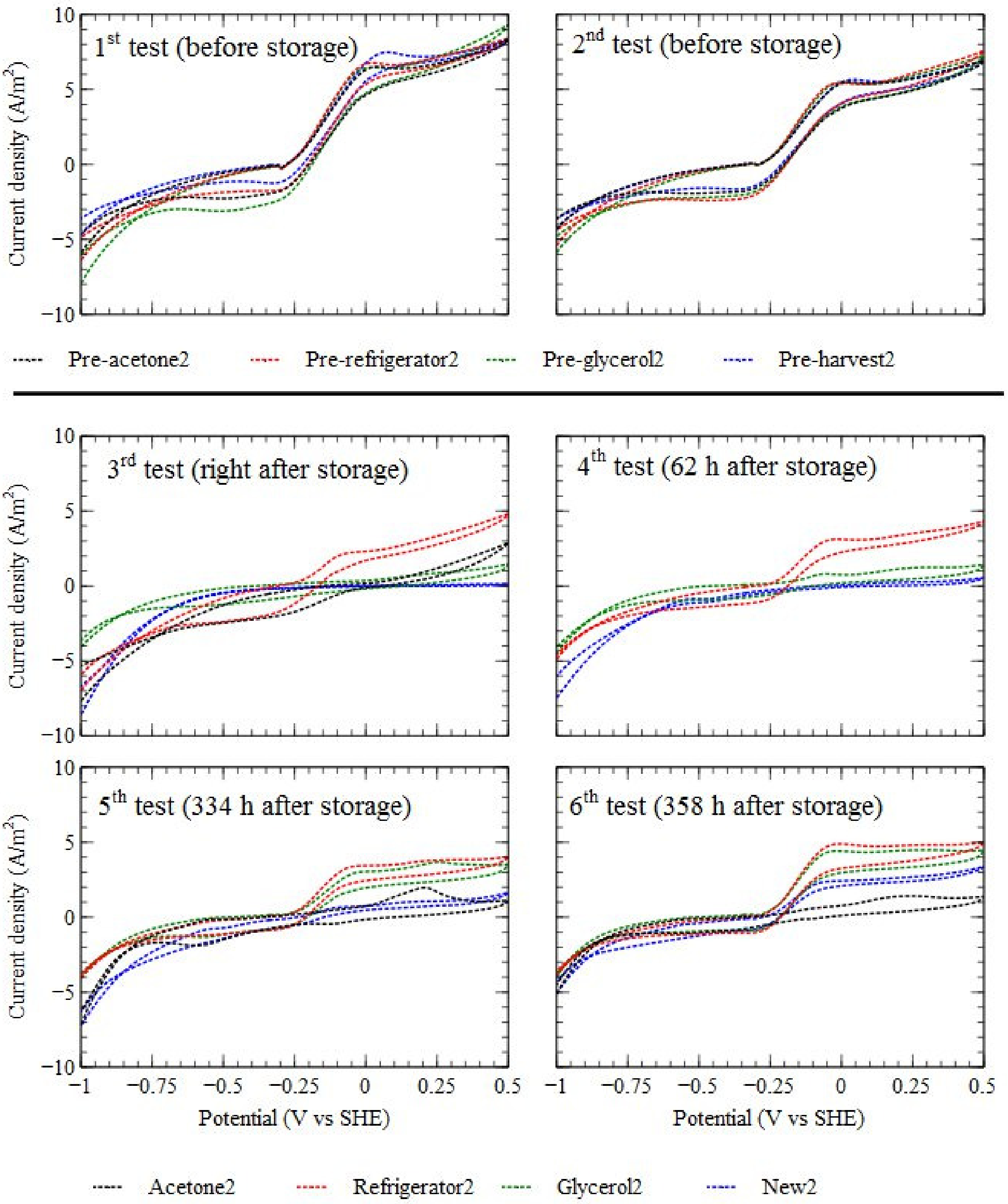

Figure S5. Cyclic voltammograms from six different tests of the anodes in the reactor. Two tests (1st-2nd) were done before storage and the rest (3rd-6th) after storage. The figure legends refer to the three storage methods used: acetone dehydration, refrigeration, and freezing in 10% glycerol solution. New refers to new graphite rod anodes placed in the reactor when the system was restarted after storage. This graph shows results from four of the eight anodes placed in the reactor (Figure S4 shows the other four).

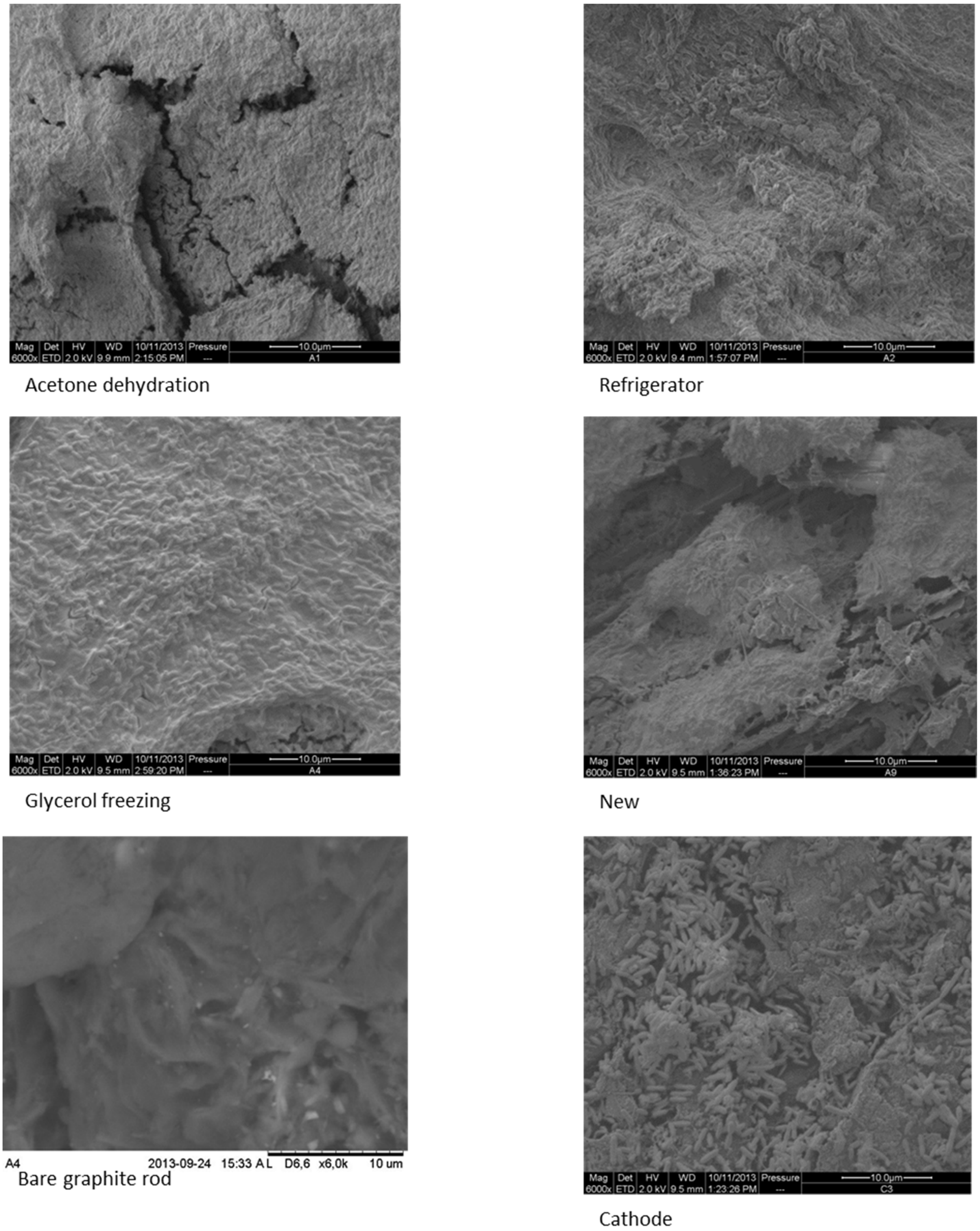

Figure S6. Scanning electron microscope images of bioanodes stored under different conditions. Images of a bare graphite rod and a cathode from the reactor are also shown. The images were taken after operating the system for 358 h after storage.

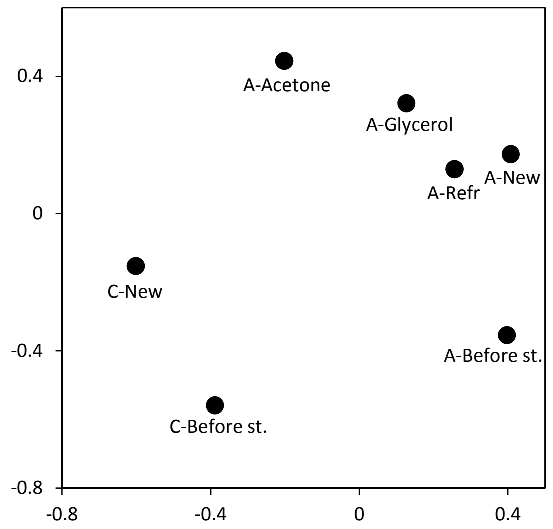

Figure S7. NMDS ordination of the microbial communities as determined by 16S rRNA gene amplicon high throughput sequencing on the anodes (A) and cathodes (C). Stress = 0.13.

Table S1. Diversity of the electrode biofilm bacterial communities based on subsampled datasets (N=2300) of the 16S rRNA gene sequences obtained by high throughput sequencing using the Illumina MiSeq platform.

Electrode	Storage method	Number of OTUs (S)	Inverse Simpson index[a]
Anodes	Before storage	117	3.6
	After storage:		
	Acetone dehydration	155	23.7
	Glycerol-freezing	154	18.7
	Refrigeration	153	11.1
	New	138	13.5
Cathodes	Before storage	82	2.0
	After storage:		
	New	158	8.0

[a] A high inverse Simpson index means high microbial diversity. The index is calculated by the following equation: $\frac{N\cdot(N-1)}{\sum_{i=1}^{S_{obs}} n_i\cdot(n_i-1)}$ where N the total number of individuals in the sample, S_{obs} is the number of observed OTUs, and n_i is the number of individuals in the ith OTU.

Supplementary methods

Nutrient medium

The nutrient medium contained per litre: 2.925 g of NaCl, 0.1 g of $CaCl_2 \cdot 2H_2O$, 0.1 g of NH_4Cl, 0.1 g of $MgSO_4 \cdot 7H_2O$, 1.65 g of $NaC_2H_3O_2$, 2.75 g of KH_2PO_4, 5.2 g of K_2HPO_4 and 0.05 g of yeast extract. 1 mL L^{-1} of trace element solution was added to the medium. The trace element solution contained (per litre): 0.05 g of H_3BO_3, 0.05 g of $ZnCl_2$, 0.03 g of $CuSO_4$, 0.5 g of $MnCl_2 \cdot 4H_2O$, 0.05 g of $(NH_4)Mo_7O_{24}$, 0.05 g of $AlCl_3$, 0.05 g of $CoCl_2 \cdot 6H_2O$, 0.05 g of $NiCl_2$, 0.1 g of Na_2SeO_3, and 0.05 g of $Na_2WO_4 \cdot 2H_2O$.

Scanning electron microscopy (SEM)

The graphite rod electrodes were prepared for SEM by fixation in 2.5% paraformaldehyde in phosphate buffer saline (PBS) at pH 7.2 overnight. The rods were then dehydrated in an ethanol series (25%, 50% and 75% for 30 min each followed by 99.5% ethanol for approximately 90 min). The dehydrated rods were rapidly cut into 12 mm pieces using a band saw, taking care not to disturb the microbial biofilms. Liquid was removed from the biofilms by submerging the 12 mm pieces in hexamethyldisilazan for two 10 min periods and then leaving them to dry overnight. Finally, the pieces were sputter-coated with gold (Quorum Q150R S) and analyzed by a FEI Quanta 200F scanning electron microscope.

PCR, sequencing, and bioinformatics

PCR was carried out using the primers 515F and 806R to amplify partial V4 region sequences of the bacterial 16S rRNA gene [1]. The primers were dual index labeled according to the approach described in Kozich, et al. [2]. The PCR reactions were carried out in duplicate reactions with 1 µl of target DNA, 1 µl of forward and reverse primers (10 µM) and 17 µl of AccuPrime Pfx polymerase SuperMix (Life Technologies). The PCR program consisted of activation (95°C, 5 min); 30 cycles of denaturation (95°C, 20 s), annealing (55°C, 15 s) and elongation (68°C, 60 s); and final elongation (68°C, 10 min). The obtained products were quality checked by standard gel electrophoresis, the duplicate PCR products were pooled and diluted to approximately 10 ng μl^{-1}. Purification was performed using the Agencourt Ampure beads technology (Beckman Coulter). The DNA concentration of the purified products was measured using the Qubit 2.0 fluorometer (Life Technologies), the PCR products were multiplexed in equimolar amounts and the pooled PCR product was diluted with Tris-Cl (pH 8.5, 0.1% Tween20) for a final concentration of 0.6 ng μl^{-1} as measured by Qubit. The expected concentration and size of the pooled PCR product was confirmed by TapeStation 2200 (Agilent Technologies). PhiX control library was spiked in at 7.5%. Sequencing was performed on an Illumina MiSeq using the MiSeq Reagent Kit v2.

The obtained sequences were processed in Mothur [3] using the MiSeq standard operating procedure for assembly of contigs, denoising, removal of putative chimera, alignment, classification and construction of operational taxonomic units (OTUs) at 97% taxonomic identity [2]. For classification with the Bayesian classifier within Mothur, the Greengenes database (v. 13.8.99, Aug. 2013) [4] was used at 80% confidence threshold. Prior to analyzing alpha and beta-diversity, the OTU dataset was subsampled at 2300 sequences. Unconstrained ordination was performed by non-metric multidimensional scaling (NMDS) [5] on You-Clayton dissimilarities [6].

Calculation of kinetic factor (K)

Electrode kinetics is usually described using the Butler-Volmer equation, which reduces to Equation S1 at low overpotentials [7].

$$i = i_0 \frac{F}{RT}(\Delta V) \qquad \text{(S1)}$$

where i is the current density, i_0 is the exchange current density, F is the Faraday constant, R is the universal gas constant, T is the temperature, and ΔV is the overpotential.

The kinetic factor ($K=i_0F/RT$) is the reciprocal of the charge transfer resistance and can be used as an index for the kinetic facility of the electrode reaction [7]. The value of K was calculated based on the linear sweep voltammetry curves for overpotentials less than 50 mV.

References

1 Caporaso, J. G. *et al.* Global patterns of 16S rRNA diversity at a depth of millions of sequences per sample. *Proceedings of the National Academy of Sciences of the United States of America* **108 Suppl 1**, 4516-4522 (2011).

2 Kozich, J. J., Westcott, S. L., Baxter, N. T., Highlander, S. K. & Schloss, P. D. Development of a dual-index sequencing strategy and curation pipeline for analyzing amplicon sequence data on the MiSeq Illumina sequencing platform. *Applied and Environmental Microbiology* **79**, 5112-5120 (2013).

3 Schloss, P. D. *et al.* Introducing mothur: open-source, platform-independent, community-supported software for describing and comparing microbial communities. *Applied and Environmental Microbiology* **75**, 7537-7541 (2009).

4 McDonald, D. *et al.* An improved Greengenes taxonomy with explicit ranks for ecological and evolutionary analyses of bacteria and archaea. *Isme J* **6**, 610-618 (2012).

5 Ramette, A. Multivariate analyses in microbial ecology. *FEMS Microbiology Ecology* **62**, 142-160 (2007).

6 Yue, J. C. & Clayton, M. K. A similarity measure based on species proportions. *Commun Stat-Theor M* **34**, 2123-2131 (2005).

7 Bard, A. J. & Faulkner, L. R. *Electrochemical methods: Fundamentals and applications*. 2nd edn, (John Wiley & Sons, Inc., 2001).
